# 19-Year Retrospective Analysis of 146 Surgically Managed Hepatic Hydatid Cysts: Complications Across Four Procedure-Based Groups

**DOI:** 10.7759/cureus.101381

**Published:** 2026-01-12

**Authors:** Sabrina Mancino, Samuel Michelini, Naomi Mancino, Roberta Civita, Marius Coros, Flavius Mocian

**Affiliations:** 1 Faculty of Medicine, "George Emil Palade" University of Medicine, Pharmacy, Science and Technology of Targu Mures, Targu Mures, ROU; 2 Faculty of Medicine, University of Siena, Siena, ITA; 3 General Surgery, Third Department of Surgery, "George Emil Palade" University of Medicine, Pharmacy, Science and Technology of Targu Mures, Targu Mures, ROU

**Keywords:** biochemical markers, hepatic hydatid cyst, liver surgery, pair procedure, postoperative complications, surgical management

## Abstract

Background: Cystic echinococcosis remains endemic in many regions, with the liver representing the most affected organ. Surgical treatment is still required for large, symptomatic, or complicated hepatic hydatid cysts.

Objective: To evaluate surgical outcomes and postoperative complications associated with different surgical approaches for hepatic hydatid cysts in a single-centre cohort over 19 years.

Methods: This retrospective observational study included 146 patients who underwent surgical treatment for hepatic hydatid cysts at Mureș Clinical County Hospital between 2004 and 2023. Patients were classified into four groups according to the surgical technique: open conservative surgery (Mabit-Lagrot, with or without Kehr drainage), minimally invasive procedures (PAIR/PAIR-D), radical or complex surgery, and ultrasound-guided drainage. Demographic, operative, and postoperative outcomes were analysed.

Results: The Mabit-Lagrot technique was the most frequently performed procedure (45.9%). Overall, postoperative complications occurred in 25.3% of patients, with recurrent abscessed hydatid cysts representing the most common event. Minimally invasive procedures were associated with shorter hospital stays and lower complication rates in selected cases. Postoperative total bilirubin and amylase levels differed significantly across surgical groups, with higher values observed after more invasive procedures.

Conclusion: Postoperative outcomes following surgical treatment of hepatic hydatid cysts vary according to procedural invasiveness. While open surgery remains effective, it is associated with higher morbidity. Minimally invasive approaches represent valid alternatives in appropriately selected patients. Early postoperative bilirubin and amylase levels appear to reflect surgical invasiveness rather than predict complications.

## Introduction

Cystic echinococcosis (CE), also known as hydatid disease, is a chronic zoonotic infection caused by Echinococcus granulosus, a tapeworm of the *Taeniidae* family [1]. Herbivorous and omnivorous animals become intermediate hosts after ingesting parasite eggs, which develop into larvae and localise within internal organs. Carnivores act as definitive hosts by ingesting infected viscera and harbouring adult tapeworms in the intestine, whereas humans are accidental intermediate hosts and do not contribute to transmission. In humans, the liver is the most affected organ (50-70%), followed by the lungs (20-30%), whereas involvement of the spleen, central nervous system, heart, kidneys, and bones is less frequent [2].

Despite global prevention efforts, the annual burden of cystic and alveolar echinococcosis exceeds one million disability-adjusted life years [3,4]. The disease often progresses silently for years, with hepatic cysts remaining asymptomatic until they reach a significant size or develop complications [5]. Clinical manifestations are primarily related to mass effect and include right upper quadrant pain, abdominal distension, and hepatomegaly. Severe or untreated cases may result in rupture, infection, cholangitis, biliary fistula, or anaphylactic shock, potentially leading to life-threatening outcomes [6].

Diagnosis is based on a combination of clinical evaluation, imaging, and serological testing. Ultrasonography is the first-line imaging modality due to its availability and reliability in cyst staging according to the World Health Organization Informal Working Group on Echinococcosis (WHO-IWGE) classification [1], with computed tomography and magnetic resonance imaging reserved for complex cases. Serological confirmation is commonly achieved using antibody-based assays, most frequently enzyme-linked immunosorbent assays (ELISA). ELISA employing the synthetic peptide p176 has demonstrated encouraging diagnostic performance, with reported sensitivity and specificity of up to 80% and 93%, respectively [7,8]. Alternative techniques, such as counter-immuno-electrophoresis and bacterial co-agglutination, are also available, although ELISA remains the preferred method for large-scale screening.

Management of hepatic hydatid cysts includes pharmacological therapy, minimally invasive techniques such as PAIR and PAIR-D, surgical interventions of varying complexity, and a watch-and-wait strategy. However, despite the availability of multiple therapeutic options, uncertainty persists regarding optimal surgical selection based on cyst characteristics and the expected postoperative outcomes. These issues are particularly relevant in endemic regions, where treatment decisions must balance efficacy, complication risk, and resource availability.

To address these issues, the present study retrospectively evaluates patients surgically treated for hepatic hydatid cysts, aiming to describe clinical and surgical characteristics and assess postoperative outcomes across different procedure-based approaches. Additionally, the study compares surgical techniques, postoperative complications, and length of hospital stay, providing insight into real-world surgical management patterns.

## Materials and methods

Study design

This retrospective observational study was conducted in accordance with the STROBE guidelines [9]. It included 146 patients diagnosed with hepatic hydatid cysts who underwent surgical treatment at Târgu Mureș Clinical County Hospital (Romania) between March 2004 and November 2023. The study was approved by the institutional Ethics Committee (Approval No. 18309). Due to the retrospective nature of the study, a waiver of informed consent was granted in accordance with institutional and ethical guidelines. Data were obtained from the General Surgery Department database.

Study objectives

The primary objective was to describe surgical outcomes and postoperative complications in patients with hepatic hydatid cysts managed using different surgical approaches over 19 years at a single centre.

Secondary objectives were to compare postoperative morbidity, recurrence patterns, operative time, and length of hospital stay across predefined surgical groups and to explore descriptive associations between early postoperative biochemical parameters (total bilirubin and serum amylase) and postoperative outcomes.

Eligibility criteria

Inclusion Criteria

All patients diagnosed with hepatic hydatid cysts confirmed by imaging (ultrasonography and/or CT scan) and/or serological testing who underwent surgical management.

Exclusion Criteria

Patients with hydatid disease without hepatic involvement, those treated exclusively with pharmacological therapy, and patients with incomplete medical records.

Data collection

Demographic data (age, sex, place of residence), clinical characteristics, type of surgical procedure, anaesthesia, operative time, postoperative course, haematological parameters (leukocyte count, platelet count, haemoglobin), and biochemical parameters (total and direct bilirubin, serum amylase) were extracted from medical records. All data were anonymised before analysis.

Because the study spans 19 years, postoperative complications were recorded as originally documented by the attending surgeons. Each complication reflected real-time clinical judgement based on the diagnostic standards routinely applied during the corresponding period. Although uniform reassessment using a single contemporary definition was not feasible, all complications were diagnosed by qualified surgeons according to the standard of care in use at the time, providing reliable real-world data for retrospective analysis.

Postoperative biochemical parameters

Postoperative total bilirubin and serum amylase levels measured within 48 hours after surgery were analysed as descriptive biochemical markers reflecting the degree of surgical invasiveness and hepatic manipulation. Values were categorised according to clinically relevant thresholds and evaluated across surgical groups.

Definition of postoperative outcomes

Postoperative complications were defined as adverse events occurring after surgery during hospitalisation or follow-up, including abdominal abscess formation, biliary leakage or fistula, abdominal effusion, and peritoneal echinococcosis.

Disease recurrence was considered a postoperative complication and was defined as the appearance of a new hepatic hydatid lesion on imaging and clinical evaluation, including abscessed or calcified cysts.

Grouping of surgical procedures

For analytical purposes, patients were categorised a priori into four groups according to the type and invasiveness of the surgical procedure. Group allocation was based on predefined criteria reflecting the surgical technique and extent of hepatic manipulation. Surgical approaches were determined by cyst characteristics, disease severity, patient condition, and surgeon judgement, rather than random assignment. This classification was applied consistently across all analyses, and the predefined groups were included in multivariable models as proxies for surgical invasiveness to limit outcome-related bias.

Group 1: Mabit-Lagrot Technique (With or Without Kehr Drainage)

This group included patients who underwent traditional open conservative surgery consisting of cyst evacuation, cavity sterilisation, and external drainage, with optional placement of a Kehr T-tube in cases of biliary communication.

Group 2: Minimally Invasive Procedures (PAIR/PAIR-D)

This group comprised patients treated with percutaneous techniques such as PAIR (puncture, aspiration, injection, re-aspiration) or PAIR-D (PAIR with instillation of a scolicidal agent), performed under ultrasound or CT guidance, and usually under local anaesthesia.

Group 3: Radical or Complex Surgical Procedures

This heterogeneous group included more extensive operations such as partial cystectomy with omentoplasty or external drainage, hepatic resections (reported using standardised terminology according to the Brisbane 2000 classification), cystogastrostomy (Pelisser technique), or surgical evacuation of abscessed cysts.

Group 4: Ultrasound-Guided Drainage

This group included patients who underwent ultrasound-guided percutaneous drainage without scolicidal injection, typically indicated for infected, suppurated, or high-risk cysts unsuitable for PAIR.

These grouping criteria were applied consistently throughout the analysis to allow comparison of postoperative outcomes across different levels of invasiveness and surgical complexity.

Statistical analysis

Statistical analyses were performed using GraphPad Prism version 10.0 (released 2023; GraphPad Software, San Diego, CA, USA). The software was used under a valid personal licence. 

Missing data accounted for less than 10% of variables. As they were missing at random, pairwise deletion was used for descriptive analyses and complete-case analysis for regression models. No imputation was performed due to the retrospective design and the small amount of missing data. Continuous variables were tested for normality using the Shapiro-Wilk test and presented as mean ± standard deviation (SD) or median (interquartile range, IQR), as appropriate. Categorical variables were reported as frequencies and percentages. Group comparisons were performed using the chi-square or Fisher’s exact test for categorical variables and the one-way ANOVA or Kruskal-Wallis test for continuous variables. When a global test indicated a statistically significant difference among groups, post hoc pairwise comparisons were performed. To account for multiple testing while preserving statistical power, the Holm-Bonferroni method was applied to adjust p-values for post hoc analyses only. No adjustment was applied to predefined global comparisons, which were limited in number and interpreted descriptively.

Binary logistic regression was used to assess predictors of postoperative complications; variables included age, sex, operative time, surgical group, and postoperative bilirubin and amylase categories. Results are reported as odds ratios (OR) with 95% confidence intervals (CI). All tests were two-tailed, and p < 0.05 was considered significant.

## Results

Patient demographics

Among the 146 patients included in this study, 80 (54.8%) were female, and 66 (45.2%) were male, with a mean age of 43.9 ± 13.2 years. The disease was most prevalent in the 30-39 age group (26%, n = 38), and most patients (72%, n = 105) came from rural areas.

Surgical groups and procedures

Patients were categorised into four groups based on surgical approach: Group one (n = 86) included Mabit-Lagrot procedures with or without Kehr drainage; Group two (n = 18) comprised minimally invasive techniques such as PAIR and PAIR-D; Group three (n = 24) included more complex or radical procedures. These consisted of partial cystectomy with drainage, standardised hepatic resections according to the Brisbane 2000 terminology [10], cysto-gastrostomy (Pelisser procedure), and abscess evacuation. A detailed breakdown of the specific techniques is provided in Table 1. Group four (n = 18) included ultrasound-guided drainage.

**Table 1:** Specific surgical procedures included in Group 3 (n = 24)

Surgical procedure type	Standard terminology (Brisbane 2000) ^[10]^	n (%)
Partial cystectomy with drainage	—	12 (50.0%)
Left hepatic lobectomy	Left hepatectomy: segment II-IV	3 (12.5%)
Cysto-gastrostomy (Pelisser)	—	2 (8.3%)
Abscess evacuation with drainage / Kehr drainage	—	7 (29.2%)
Total	—	24 (100%)

The Mabit-Lagrot procedure was the most frequently employed (45.9%, n = 67), followed by minimally invasive techniques (12.3%, n = 18). Radical or complex surgeries were less common (16.4%, n = 24). A significant association was observed between the surgical technique and anaesthesia type (p < 0.001), with general anaesthesia predominantly used for invasive procedures (100% in Group one and 95.8% in Group three), and local anaesthesia more common for minimally invasive or percutaneous interventions (16.7% in Group two and 27.8% in Group four). The mean age was comparable across groups (ranging from 40.2 to 42.2 years), with no statistically significant differences in age or gender.

Operative time

Operative time varied significantly among groups (p < 0.001), with longer durations observed in cases requiring more extensive intraoperative management. Group one averaged 1.96 hours, Group three 2.05 hours, Group two 3.33 hours, and Group four 2.5 hours. Operative time exhibited a non-normal distribution (Shapiro-Wilk p < 0.001); thus, a Kruskal-Wallis test was applied. A statistically significant difference was found across the four groups (H = 22.8, p < 0.001).

Post-hoc pairwise comparisons using Dunn’s test with Holm-Bonferroni correction showed that Group 1 had significantly shorter operative times compared with Group 2 (p = 0.004) and Group 4 (p = 0.031). Group 2 demonstrated substantially longer operative times when compared with Group 3 (p = 0.002). Group 3 had shorter operative times compared with Group 4 (p = 0.048). The Group 1 procedures had the shortest operative time, while Group 2 procedures exhibited the longest operative time, significantly exceeding that of several other groups.

Hospital stays

Hospital stay was also influenced by the type of surgery (p = 0.001); shorter hospitalisations (<10 days) were common in Groups two (88.9%) and four (77.8%), whereas prolonged stays (10-19 days) occurred mainly in Group one (58.1%), consistent with the invasiveness of the technique. Prolonged hospitalisation (>20 days) was uncommon across the study groups. No patients in Group 2 experienced a hospital stay longer than 20 days, whereas prolonged hospitalisation was observed in 14 patients in Group 1 (16.3%), two patients in Group 3 (8.3%), and one patient in Group 4 (5.6%). Hospital stays also showed a non-normal distribution (Shapiro-Wilk p < 0.001), and the Kruskal-Wallis test demonstrated a significant difference between groups (H = 19.6, p < 0.001).

Post-hoc Dunn tests with Holm-Bonferroni adjustment identified the following significant contrasts: Group 1 vs. Group 2: p = 0.014, Group 1 vs. Group 4: p = 0.040, Group 3 vs. Group 2: p = 0.022.

Postoperative hematologic and biochemical outcomes

Haematologic evaluation revealed postoperative leukocyte abnormalities in 21.9% of patients in Group one, while no cases were reported in Group two. Leukopenia was rare (3.4%) and mainly found in Group four. Thrombocytopenia (6.2%) and thrombocytosis (5.5%) were mild and not statistically significant. Biochemical parameters showed significant postoperative variations in total bilirubin (Table 2) and serum amylase (Table 3). Early postoperative total bilirubin and serum amylase levels differed significantly between the surgical groups. Higher postoperative bilirubin values were more frequently observed after open and complex procedures, while amylase elevations were also more common in patients undergoing more invasive surgeries.

**Table 2:** Association between postoperative total bilirubin levels and surgical procedure groups ^a^Fifteen cells (62.5%) have expected counts less than 5. The minimum expected count = 0.12. bp-value corresponds to the Pearson Chi-square test with Monte Carlo correction applied. Monte Carlo significance values are based on 10,000 sampled tables (seed: 205597102)

Postoperative total bilirubin (mg/dL)	Group 1 (Mabit-Lagrot)	Group 2 (PAIR/ PAIR-D)	Group 3 (radical/ complex)	Group 4 (Echo-guided drainage)	Total (n=146)	p-values	Statistical analysis
>5	1 (1.2%)	0 (0.0%)	1 (4.2%)	0 (0.0%)	2 (1.4%)	.036^b^	Pearson Chi-Square = 27.185^a^; df = 15; p = 0.027 (Monte Carlo: 0.036; 95% CI: 0.032–0.040)
1.2–2.0	5 (5.8%)	2 (11.1%)	2 (8.3%)	0 (0.0%)	9 (6.2%)	.028^b^	Likelihood Ratio = 24.396; df = 15; p = 0.059 (Monte Carlo: 0.028; 95% CI: 0.025–0.031)
2.01–3	1 (1.2%)	0 (0.0%)	1 (4.2%)	0 (0.0%)	2 (1.4%)	.016^b^	Fisher’s Exact Test = 24.679; p = 0.016 (Monte Carlo: 0.016; 95% CI: 0.013–0.018)
3.01–4	0 (0.0%)	1 (5.6%)	0 (0.0%)	0 (0.0%)	1 (0.7%)		
No data	25 (29.1%)	8 (44.4%)	5 (20.8%)	13 (72.2%)	51 (34.9%)		
Normal values	54 (62.8%)	7 (38.9%)	15 (62.5%)	5 (27.8%)	81 (55.5%)		
Total (per group)	86 (100%)	18 (100%)	24 (100%)	18 (100%)	146 (100%)		

**Table 3:** Association between postoperative serum amylase levels and surgical procedure groups ^a^Four cells (33.3%) have expected counts less than 5. The Minimum expected count = 0.99. ^b^p-value corresponds to the Pearson Chi-square test with Monte Carlo correction applied. Monte Carlo significance values are based on 10,000 sampled tables (seed: 205597102)

Postoperative Serum Amylase (U/L)	Group 1 (Mabit-Lagrot)	Group 2 (PAIR/ PAIR-D)	Group 3 (Radical/ Complex)	Group 4 (Echo-guided drainage)	Total (n=146)	p-values	Statistical Analysis
No data	36 (41.9%)	9 (50.0%)	10 (41.7%)	16 (88.9%)	71 (48.6%)	
Normal values	46 (53.5%)	7 (38.9%)	13 (54.2%)	1 (5.6%)	67 (45.9%)	.013^b^	Pearson Chi-Square = 16.295^a^; df = 6; p = 0.012 (Monte Carlo: 0.013; 95% CI: 0.011–0.015)
Pathologically elevated values	4 (4.7%)	2 (11.1%)	1 (4.2%)	1 (5.6%)	8 (5.5%)	.005^b^	Likelihood Ratio = 18.779; df = 6; p = 0.005 (Monte Carlo: 0.005; 95% CI: 0.004–0.006)
Total (per group)	86 (100%)	18 (100%)	24 (100%)	18 (100%)	146 (100%)	.003^b^	Fisher’s Exact Test = 18.080; p = 0.003 (Monte Carlo: 0.003; 95% CI: 0.002–0.004)

Postoperative complications and recurrence patterns

Postoperative outcomes varied significantly across groups. Overall, 92% of patients had favourable recoveries, and complications occurred in 25.3% (n = 37) of cases. The most frequent postoperative complication was disease recurrence, presenting as an abscessed hydatid cyst (54.1%). In Group 3, this complication occurred in 7 out of 24 patients: five (71.4%) had undergone abscess evacuation with drainage, and two (28.6%) had undergone partial cystectomy with drainage. No abscessed hydatid cyst occurred in the three patients who underwent left hepatic lobectomy or the two patients who underwent cysto-gastrostomy (Pelisser procedure). Abdominal effusion was mainly observed in Groups two and four (66.7% each). At the same time, recurrent calcified hydatid cysts were reported in groups one (n=2) and two (n=1), and a single case of peritoneal echinococcosis occurred after open surgery. A comprehensive summary of all complications stratified by surgical group is presented in Table 4.

**Table 4:** Associations between postoperative complications and recurrence patterns according to surgical procedures ^a^Four cells (50.0%) had expected counts less than five. The Minimum expected count =1.38. ^b^p-value corresponds to the Pearson Chi-square test with Monte Carlo correction applied (random seed: 205597102). Holm‑adjusted pairwise comparisons: Group 2 vs. Group 4: p = 0.004; Group 1 vs. Group 3: p = 0.033; Group 1 vs. Group 4: p = 0.028

Postoperative complications and recurrence patterns		Group 1 (Mabit-Lagrot)	Group 2 (PAIR/ PAIR-D)	Group 3 (radical/ complex)	Group 4 (Echo-guided drainage)	Total	p-value	Statistical analysis
Recurrent abscessed hydatid cyst	Count	8	0	7	5	20	.005	Pearson Chi-Square = 23.21^a^; df = 9; p = 0.0057 (Monte Carlo: 0.0317; 95% CI: 0.0283 – 0.0351)
							.0011	Likelihood Ratio = 27.59; df = 9; p = 0.0011 (Monte Carlo: 0.0029; 95% CI: 0.00185– 0.00395)
	% within groups	66.7%	0.0%	100.0%	33.3%	54.1%		
Abdominal effusion after cyst evacuation	Count	1	2	0	10	13		
	% within groups	8.3%	66.7%	0.0%	66.7%	35.1%		
Recurrent calcified hydatid cyst	Count	2	1	0	0	3		
	% within groups	16.7%	33.3%	0.0%	0.0%	8.1%		
Peritoneal echinococcosis	Count	1	0	0	0	1		
	% within groups	8.3%	0.0%	0.0%	0.0%	2.7%		
Total	Count	12	3	7	15	37		
	% within groups	100.0%	100.0%	100.0%	100.0%	100.0%		

## Discussion

This large single-centre retrospective analysis describes the clinical characteristics, surgical techniques, and postoperative outcomes of patients treated for hepatic hydatid cysts. The demographic profile of our cohort is consistent with previously published series, showing a predominance of middle-aged female patients from rural areas in endemic regions [11]. The clear predominance of rural patients (72%) highlights the strong association between echinococcosis and livestock exposure in endemic areas. The association between rural and urban residence confirms that direct contact with infected animals and farming activities remain key risk factors for transmission.

Comparison of surgical approaches

The optimal surgical approach for hepatic hydatid disease remains a matter of debate. While some authors support radical surgery for its lower risk of bile leakage and recurrence, others argue that such interventions expose patients to unnecessary risks in the management of a benign condition, favouring conservative procedures instead [12-15]. In our study, 24 patients (16.4%) underwent a radical/invasive surgical procedure (Group three). This group showed a lower incidence of postoperative complications (18.9%) compared with patients treated with conservative surgery, in whom complications occurred in 32.4%. These findings are in line with previously published retrospective series by Kulan B. Deo, Ganga R. Verma, et al. In their cohort, 27 out of 64 patients underwent radical surgery, with a reported complication rate of 7.4%, compared with 27.0% among those treated with conservative procedures [12]. However, direct comparisons between surgical approaches should be interpreted cautiously, as differences in patient selection, cyst complexity, and disease severity may substantially influence postoperative outcomes. Importantly, patients undergoing radical or complex surgery in our cohort likely presented with more advanced or complicated disease at baseline. Therefore, differences in postoperative outcomes across surgical groups should be interpreted as associations influenced by disease severity and surgical indication rather than evidence of procedural superiority. Two patient groups underwent minimally invasive procedures: Group two and Group four included 18 patients each, but the complication rates differed significantly; only three patients (8.1%) in Group two experienced complications, compared with 15 patients (40.5%) in Group four, in contrast to the published literature [16]. A more detailed description of the complications observed in the different groups is presented in Table 4.

Hospital stay and operative time

Minimally invasive procedures were associated with the longest operative times. Nevertheless, they resulted in the shortest hospital stays, likely reflecting their technical complexity coupled with reduced surgical trauma and faster postoperative recovery. In contrast, Group 1 procedures required shorter operative times but were associated with slightly longer hospitalisation, possibly due to their relatively more invasive nature and the need for extended postoperative monitoring. These findings underscore that longer surgery does not necessarily translate into a longer hospital stay and support the selective use of minimally invasive techniques to optimise recovery and resource utilisation. Patients in Group 3, who underwent major hepatic surgeries and abscess evacuations, experienced the longest hospital stays (>20 days), consistent with literature reporting shorter hospitalisation after laparoscopic procedures (mean 5.4 days) compared with open surgery (mean 8.8 days) [17].

Overall, these findings highlight that surgical invasiveness, anaesthesia type, and postoperative enzyme dynamics strongly influence recovery and complication profiles. Open approaches, such as Mabit-Lagrot, remain reliable and effective, providing stable outcomes with manageable morbidity. Minimally invasive or percutaneous methods, despite shorter hospital stays, require careful monitoring due to the potential risk of underrecognised complications, such as effusions or enzymatic leakage. However, more extensive procedures or open approaches may still be necessary depending on the clinical scenario.

Postoperative laboratory parameters

Postoperative laboratory analyses included complete blood count (haemoglobin), inflammatory markers (leukocyte and platelet counts), and biochemical parameters (blood amylase and total bilirubin) to assess surgical invasiveness and physiological response to hepatic manipulation. Leukocytosis was observed in a subset of patients, primarily reflecting secondary infection. Platelet counts were mostly within normal ranges, though mild deviations occurred in some cases, suggesting their potential utility in identifying systemic inflammation or hypersplenism related to portal hypertension. Haemoglobin abnormalities were also noted, with a spectrum ranging from mild to severe anaemia.

Bilirubin and amylase levels demonstrated notable postoperative changes, more frequently in patients undergoing open or complex procedures. Elevations in bilirubin were more common in Group 1, while significant increases were occasionally observed in Groups 1 and 3. Abnormal amylase values were predominantly detected in Group 1. These patterns are concordant with previously reported postoperative laboratory trends from Nepal [12].

In our cohort, bilirubin and amylase served as indicators of surgical complexity. Effective management relies on thorough evacuation of cyst contents with minimal spillage, sterilisation of the cavity, and secure closure to prevent recurrence and complications, including invasion of adjacent structures, suppurative infections, rupture into the peritoneal cavity or bile ducts, and mass effect-related comorbidities [18-20].

Postoperative complications

Although external biliary fistula is consistently reported as the most common postoperative complication after surgery for hepatic hydatid cysts [21-26], abscess formation was the predominant adverse outcome in our cohort. This finding likely reflects the complexity of treated cases and the degree of intraoperative contamination, particularly in patients undergoing more aggressive or extensive procedures, supporting the association between increased surgical invasiveness and postoperative infectious complications.

Abdominal effusion occurred predominantly after percutaneous or ultrasound-guided interventions, suggesting a stronger peritoneal inflammatory response in these settings and emphasising the importance of meticulous fluid control and peritoneal protection. Careful inspection of the cyst cavity and surrounding structures remains essential to ensure adequate closure and reduce postoperative morbidity. Minimally invasive approaches, including laparoscopy, offer the advantage of direct visualisation, facilitating the identification of biliary leaks and residual communications.

Conservative open procedures, such as the Mabit-Lagrot technique, were frequently associated with late cyst calcification and secondary suppuration. While effective for chronic or inactive cysts, these techniques may not fully prevent secondary infection. In hydatid disease, cyst calcification more likely represents late disease evolution or incomplete parasitic eradication rather than an acute postoperative event and should therefore be interpreted as delayed recurrence.

A rare case of peritoneal echinococcosis following ultrasound-guided drainage was observed, possibly due to intraoperative spillage or cyst rupture, underscoring the need for careful patient selection and procedural execution. Consistent with previous reports, PAIR and PAIR-D techniques were associated with more favourable postoperative outcomes than open surgery, supporting their role as safe and effective options for appropriately selected, uncomplicated hepatic hydatid cysts [27-30].

Limitations of the study

This study has several limitations. Its retrospective design may have introduced information and selection bias over nearly two decades. Being a single-centre experience, the findings may not be fully generalisable. Surgical techniques were chosen based on cyst characteristics, disease severity, clinical indication, and surgeon judgement, resulting in baseline heterogeneity, with radical or complex procedures often applied to more advanced cases. No formal adjustment for confounders was performed, so observed differences should be interpreted as associations rather than causal effects. Missing or incomplete data may have limited some clinical, laboratory, and follow-up variables, and treatment-related biases may have influenced both surgical choice and complication rates. Despite these limitations, this study provides valuable insights from one of the largest cohorts of surgically treated hepatic hydatid cysts and may inform future prospective multi-centre studies with standardised surgical criteria and severity stratification. Further research is warranted to further validate these findings.

## Conclusions

The surgical management of hepatic hydatid cysts encompasses a wide spectrum of techniques that should be tailored to cyst characteristics and disease severity. In this single-centre retrospective cohort, minimally invasive approaches were associated with shorter hospital stays and lower postoperative morbidity in selected patients, while open and complex procedures remained necessary for advanced or complicated disease. Postoperative variations in bilirubin and amylase levels appeared to reflect the degree of surgical invasiveness rather than predict adverse outcomes. These findings support an individualized approach to treatment selection and highlight the need for future prospective studies to better define optimal management strategies.
